# Demethylzelasteral inhibits proliferation and EMT via repressing Wnt/β-catenin signaling in esophageal squamous cell carcinoma

**DOI:** 10.7150/jca.45493

**Published:** 2021-05-10

**Authors:** Jiarui Yu, Wei Wang, Baolin Liu, Jinling Gu, Siyuan Chen, Yishuang Cui, Guogui Sun

**Affiliations:** 1 School of clinical medicine, Affiliated Hospital, School of Public Health, North China University of Science and Technology, Tangshan, Hebei 063000, China.; 2 Department of Radiation Oncology, North China University of Science and Technology Affiliated People's Hospital, Tangshan, Hebei 063000, China.

**Keywords:** Demethylzeylasteral, T-96, ESCC, proliferation, cell cycle, apoptosis, EMT, Wnt/β-catenin pathway

## Abstract

As a kind of tumor commonly seen, no effective treatment is available for esophageal squamous cell carcinoma (ESCC). Therefore, seeking a new treatment is urgent. Demethylzeylasteral (T-96) isolated from Tripterygium wilfordii root bark embraces outstanding good antitumor activity. However, as for the mechanism of T-96 work on ESCC cells, it is rarely reported. In this study, we found that T-96 has inhibition when ESCC cells are proliferating, migrating and cloning. Moreover, relevant effects are influenced by dose and time. And T-96 can result in the stop of G2/M phase and induce apoptosis of ESCC cells. In addition, the expressions of Cyclin B1, Cyclin D1, Bcl-2, PARP1 and Survivin were decreased after starch demethylation. Despite of this, Bax and PARP1's expressions went up. To add up, there was an obvious increase in the expression of E-cadherin, while that of N-cadherin, Vimentin and MMP9 decreased after T-96 treatment. Moreover, the expression of Wnt/β-Catenin pathway, which concerns proteins β-Catenin, c-Myc and Wnt3a decreased. Our study shows that T-96 inhibits the proliferation and migration of esophageal cancer cells through Wnt/β-catenin pathway. Moreover, it gives rise to cell cycle arrest and apoptosis. According to the research results, T-96 tends to be put into use when treating ESCC patients, thus laying the experimental foundation for clinical research.

## Introduction

Esophageal squamous cell carcinoma (ESCC) is a common type of esophageal cancer in the world, and its incidence is very high in China. To date, esophageal cancer is the seventh most common type of cancer and the sixth leading cause of cancer-related deaths worldwide 1. Surgical resection is only applicable to who have suffered from the cancer in the early stage. Unfortunately, esophageal lesions tend to be neglected, thus resulting in a delay when treating it 2, 3. Advanced esophageal cancer is prone to metastasis, and is difficult to be cured by surgery. Chemotherapy turns out to be significant in cancer treatment, but the current chemotherapeutics are still unable to achieve satisfactory results 4. Given that esophageal cancer cells exhibit drug resistance, developing effective chemotherapy drugs is urgently needed and will be beneficial for the cancer treatment.

Demethylzeylasteral (T-96), as an effective component, it was isolated from Tripterygium wilfordii in the 1990s. This compound is supposed to be able to fight against inflammatory and tumor effects 5. T-96 displays significant anticancer activities in various human malignancies, including glioma, melanoma, breast, and pancreatic cancer 6-9. Based on several studies, T-96 is able to attenuate the growth of cells, affect the tumor cell cycle, promote apoptosis, and inhibit metastasis. However, it remains to be investigated whether T-96 has antitumor effects in ESCC. Therefore, we have decided to study T-96's effects on ESCC cell proliferation and migration. In the meantime, we are going to study more about the systems behind. This study could improve the comprehension of the biological relevance of T-96 in ESCC, thus resulting in its better use.

It is known that the Wnt/β-catenin signaling should not be excluded in the whole process of cell growth, metastasis, and apoptosis. To add up, it is connected with epithelial-mesenchymal-transition (EMT) 10, 11. As a transmembrane protein, N-cadherin (N-cad) functions in cell-to-cell adhesion, while E-cadherin (E-cad) is required for forming intercellular connections.Loss of E-cadherin may lead to enhanced tumor infiltration and metastasis 12, 13. β-catenin is critical of Wnt signaling pathway, and the inactivation during this process might result in the development of cancer 14. When carrying out the study, it is found out that T-96 is able to slower the pace of cell proliferation, migration and EMT. Meanwhile, through regulations of pathway, it manages to enhance apoptosis. This study revealed an anti-tumor potential of T-96 when treating the cancer.

## Materials and Methods

### Reagents, antibodies and kits

Purchased reagents, antibodies and kits: Demethylzeylasteral (T-96) from Sellck (Selleckchem, Houston, USA), antibodies against N-cadherin, E-cadherin, Vimentin, MMP9, Bcl-2, GAPDH, Wnt3a and β-actin from Proteintech (Wuhan, China), antibodies against Survivin, Bax, cyclinD1, β-catenin and c-Myc from Cell Signaling (Danvers, MA, USA), Cell Counting Kit-8 (CCK8) from Beyotime Biotechnology (Shanghai, China). The kit for detecting cell cycle and FITC-conjugated Annexin V kit for detecting apoptosis from NEOBIOSCINECE (Shenzhen, China).

### Cell culture and treatments

The KYSE150 and KYSE410 cell lines came from the laboratory of Professor Hirotaka Shimada at Kyoto University, Japan. ESCC cells were cultured in RPMI 1640 medium containing 10% FBS, 100 U/mL penicillin, and 100 μg/mL streptomycin, and humidified with 5% CO_2_ in an incubator at 37 °C.

### Cell viability assay

Cell viability was measured by Cell Counting Kit-8 (CCK8). ESCC cells in logarithmic growth phase were collected and evenly seeded on three 96-well plates (3×10^3^ cells/well). After being cultured for 24 hours, T-96 with 0, 5, 10, 20 concentrations (each concentration of 6 sub pores) was cultured for 24, 48, 72 hours. Then the cells were coated with medium of CCK8 and cultured in dark for 2 hours. At last, the microplate reader is used to measure the absorbance at 450 nm. Each experiment was carried out three times, and the average value was regarded as results.

### Colony formation assay

The logarithmic growth ESCC cells were evenly seeded into three 6-well plates. After incubation for 24 hours, 0, 5, 10 μM T-96 were coated on the plate for 12 hours. After centrifugation, 400 cells were cultured in normal medium and subcultured in a 12-well plate. 14 days later, the viable colonies were fixed in 100% methanol and stained with 1% crystal violet for 10 minutes. In the end, the plate will be cleaned with tap water for 3 times and dried at RT. When carrying out the study, the experiment was repeated three times.

### Transwell assay for cell migration

ESCC cells grew logarithmically on a 6-well plate. After 24 hours of culture, 0, 5, and 10 μM T-96 were coated on the growing cells. The cells would consequently grow for 12 hours, then are digested and counted. 100,000 live cells were re-suspended in serum-free medium and inoculated evenly into the upper cavity. A 700 μL culture medium containing 30% FBS was then injected into each lower half chamber. After 24 hours, soak in precooled methanol for 10 minutes, and then dye with 0.5% crystal violet, which lasted 10 minutes. The migrated cells were photographed and quantitatively analyzed with a microscope. The experiment was carried out three times.

### Cell cycle analysis

ESCC cells were treated with T-96 (0, 5 and 10 μM) and they are of different concentrations. After 48 hours, cells were collected, cleaned with PBS, and fixed overnight with 70 % ethanol ice at 4 °C. The cells were stained with propidium iodide (PI) solution for 30 minutes. Flow cytometry was used to detect DNA content (BD Biosciences, NJ, USA). Each group was repeated three times.

### Cell apoptosis analysis

Apoptosis was detected by flow cytometry using Annexin V-FITC kit (NEOBIOSCINECE, Shenzhen, China). In short, ESCC cells were cultured in 0, 5, or 10 μM T-96 for 48 hours and then examined morphologically under Leica DMI4000B microscope (Leica Microsystems GmbH, Wetzlar, Germany). Adherent cells and floating cells were treated with trypsin and incubated with 5 μL of FITC-conjugated annexin V (0.5 mg/mL) for 15 minutes, followed by incubation in the dark with 5 μL of FITC-conjugated annexin V (0.5 mg/mL) for another 15 minutes. This experiment used BDTM LSRΙΙ flow cytometry instrument (BD Biosciences, NJ, USA) when detecting annexin positive cells apoptosis cells. For each group, the experiment was conducted three times.

### RNA extraction and qRT-PCR

Total RNA managed to be extract through TRIzol reagent (Invitrogen, USA) based on relevant guidance 15. Synthesis of cDNA was carried out through PrimeScript™ RT reagent Kit with gDNA Eraser (TaKaRa, Kusatsu, Japan). Real-timePCR got managed by CFX Connect Real-time PCR system (BioRad) using SYBR Premix Ex Taq kit (TaKaRa). The primers mentioned are shown in Table 1.

### Western blot analysis

ESCC cells were incubated with T-96 (0, 5 or 10 μM) for 24 hours. After having been cleaned with PBS twice, the cells were digested and centrifuged with trypsin, and then the total proteins were extracted. The protein content was up to plyl-BCA protein quantitative kit. Equivalent protein samples were separated through SDS-PAGE gel electrophoresis and moved to PVDF membrane (Millipore, USA). Cell membranes were closed with 5% skimmed milk or 2% bovine serum albumin (BSA). Later, they were incubated with primary and corresponding secondary antibody. The target protein in the membrane was detected and observed with the aid of chemiluminescence kit. The main antibodies used in the experiment are as follows: E-cadherin, N-cadherin, Vimentin, MMP9, Bcl-2, β-actin, Survivin, Bax, cyclinD1, Cyclin B1, β-catenin, Wnt3a and c-Myc. The experiment was performed thrice.

### Statistical analysis

All data were analyzed with GraphPad Prism 8.0 and the quantitative data were expressed as means±SD. One-way ANOVA was used to analyse the difference of two groups. Under all circumstances, if *P* < 0.05, then significant difference existed.

## Results

### An inhibition of T-96 on ESCC cell proliferation

**Figure 1:**
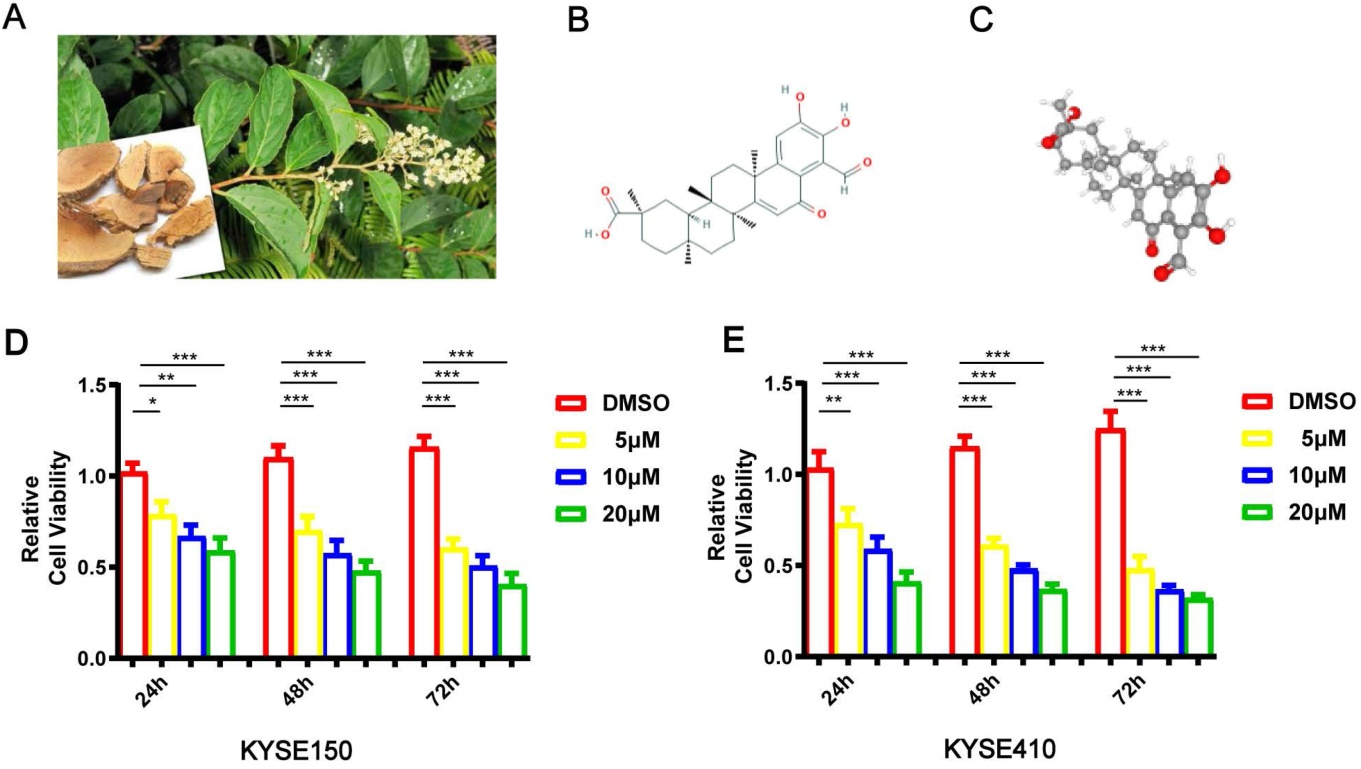
Demethylzeylasteral(T-96) inhibits the proliferation of ESCC cells. (A) Leaves and roots of Chinese herbal medicine Tripterygium wilfordii. (B) Chemical structure of Demethylzeylasteral. (C)3D Conformerstructure of Demethylzeylasteral. (D-E) After the KYSE150 and KYSE410 cells were treated with 24, 48 and 72 hours with Demethylzeylasteral of different concentration gradients (0, 5, 10 and 20 µM), CCK8 assay was used to detect the viability of the ESCC cells. Data shown are means ± SEM from 3 independent experiments in duplicate. *P < 0.05, **P < 0.01,* P < 0.001 as compared to the control group.

The pictures of Tripterygium wilfordii and its roots are shown in Figure 1A. Demethylzeylasteral (T-96)'s chemical and 3D structures are shown in Figure 1B-C. CCK8 was carried out when studying T-96's effects on esophageal cancer cells' viability. In pursuit of this goal, KYSE150 and KYSE410 cells were incubated with T-96 (0, 5, 10 or 20 μM) for 24 hours, 48 hours and 72 hours, respectively. The chemical structural formula of T-96 can be found out in Figure 1D. According to the cell viability test, treatments with T-96 could result in inhibitions, which were influenced by dose and time (Figure 1E).

### T-96 inhibits the cancer cell migration and colony formation in ESCC

**Figure 2:**
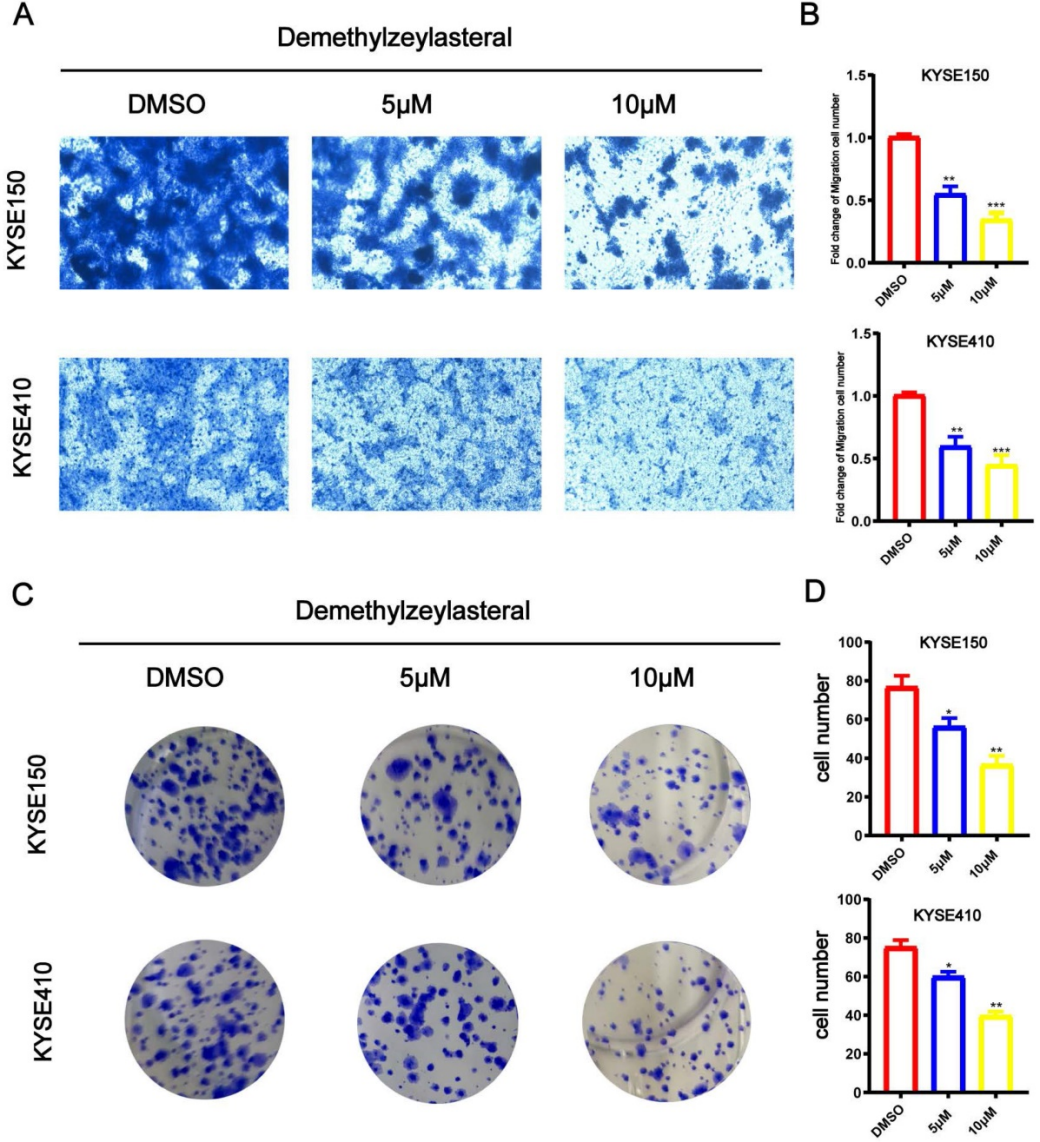
Demethylzeylasteral (T-96) inhibits the migration and colony formation of ESCC cells. The effects of different concentrations of Demethylzeylasteral on ESCC migration were determined by Transwell assay. (A) Demethylzeylasteral treatment reduced the migration capacity of KYSE150 and KYSE410 cells in a dose-dependent manner. (B) Bar chart of number of migrating cells. (C) ESCC cells clone formation ability. Demethylzeylasteral reduced colony formation in KYSE150 and KYSE410 cells in a dose-dependent manner. (D) Bar chart of number of colony formation cells.Data shown are means ± SEM from 3 independent experiments in duplicate. * P<0.05, ** *P* <0.01, *** *P* <0.001 as compared with the control group.

When trying to study influences of T-96 on the biological function of KYSE150 and KYSE410 cell migration, Transwell analysis was carried out. It is demonstrated that when KYSE150 and KYSE410 cells were dealt with T-96 (0, 5 or 10 μM), compared with the control group, the number of migrating cells was fewer (Figure 2A), indicating that T-96 was dose dependent. It is proved that as the concentration of T-96 increased, the colony forming ability decreased significantly (Figure 2B).

### T-96 induces cell cycle arrest in ESCC cells

**Figure 3:**
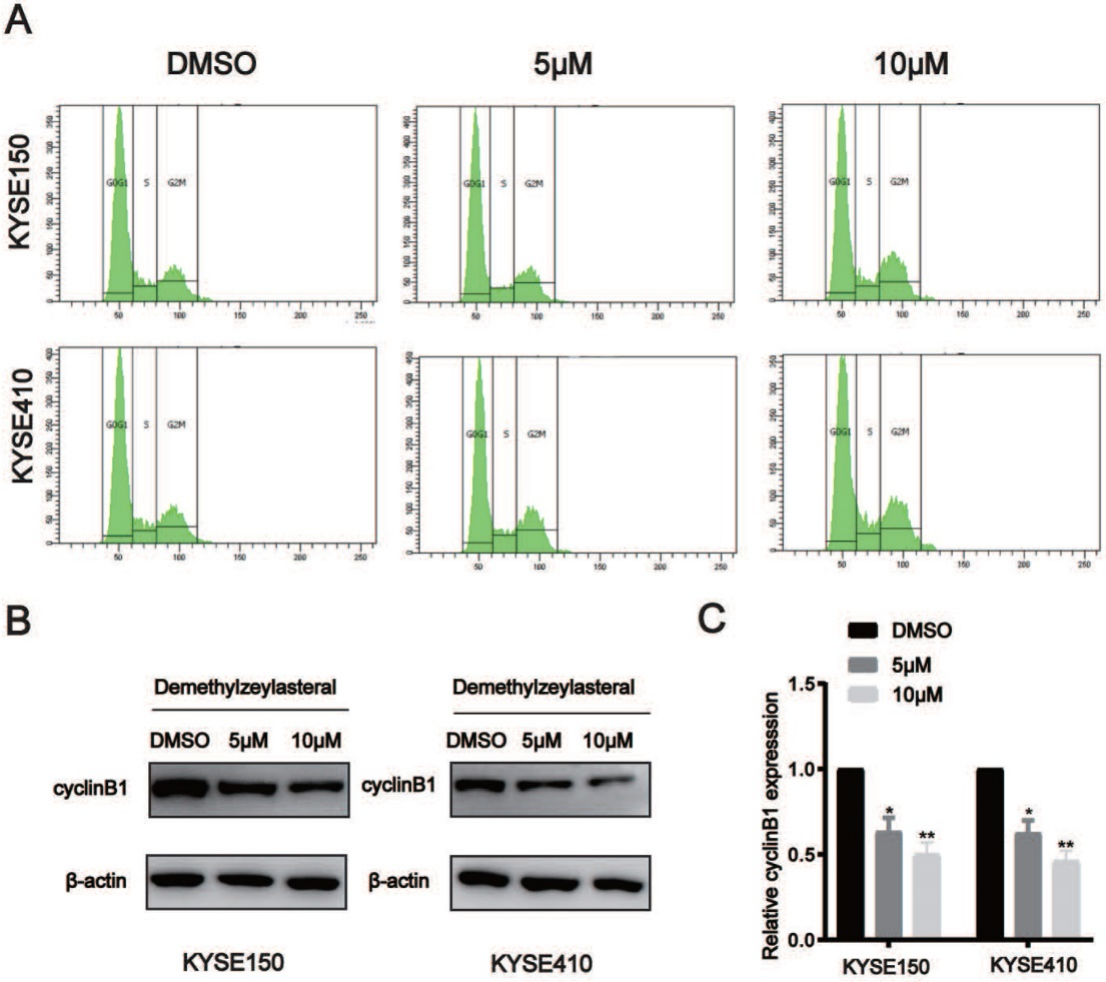
Demethylzeylasteral (T-96) causes cell cycle arrest at G2/M phase in ESCC cells. (A) Representative results of cell cycle distribution. After a 48 hours exposure to osthole at concentrations of 0, 5 and 10 µM, the cell cycle distributions of KYSE150 and KYSE410 cells were measured by flow cytometry with PI staining. (B)The CyclinB1 was detected by Western blot. (C) The protein levels of cyclinB1 was measured by western blotting. β-actin was served as loading control.*P < 0.05, **P < 0.01 as compared to the control group.

When telling T-96's effects on ESCC cells' cell cycle, flow cytometry analysis was adopted. When making a comparison, cells exposed to T-96 at 5 and 10 μM concentration will result in the aggregation of a large amount of G2/M, and the population of G0/G1 reduced accordingly (Figure 3A). In order to study whether the potential mechanism of G2/M phase arrest is related to T-96 treatment, the effect of T-96 on the expression of CyclinB1 was tested, which turns out to be critical As expected, the expression of CyclinB1 protein and mRNA in KYSE150 and KYSE410 cells treated with T-96 decreased (Figure 3B-C). Based on results, it could be claimed that through affecting the cell cycle process, T-96 managed to inhibit ESCC cells' prolife.

### T-96 induces apoptosis in ESCC cells

**Figure 4:**
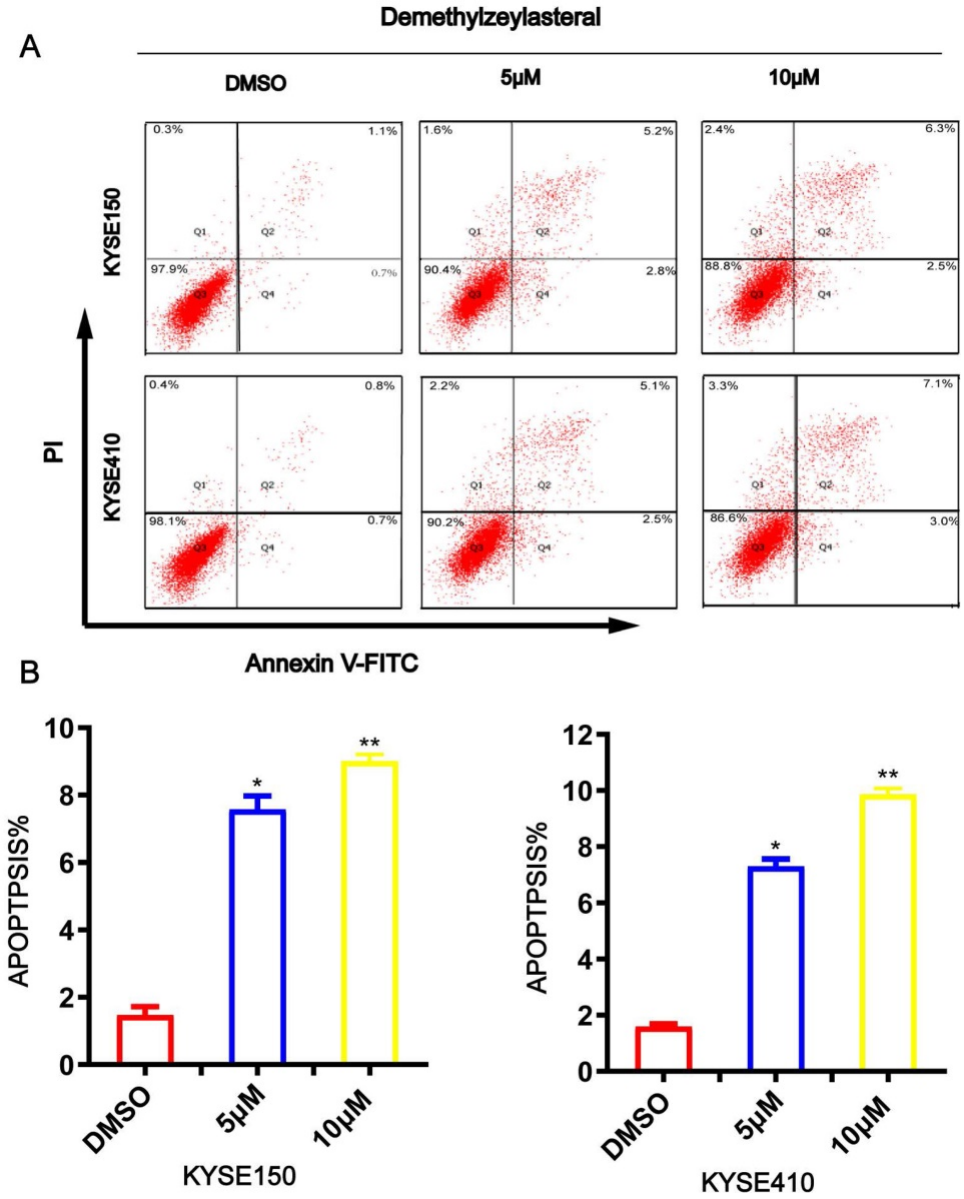
Demethylzeylasteral(T-96) promotes apoptosis in ESCC cells. (A) Apoptosis was assessed based on Annexin V-FITC/PI dual staining and flow cytometry. (B) Representative results are shown and the percentage of apoptotic cells is plotted.* *P* <0.05, ** *P* <0.001 as compared to the control group.

To evaluate whether t-96 induces ESCC cell apoptosis, Annexin V/PI was used to stain ESCC cells and flow cytometry analysis was performed. As shown in Figure 4A and 4B, an increase in T-96 concentration led to an obvious increase in the proportion of apoptotic cells in the cancer cell line. The apoptotic rates of KYSE150 and KYSE410 cells were 1.47 ± 0.25% and 1.6 ± 0.1% respectively in untreated groups, while they were 8 ± 0.62% and 7 ± 0.26% in cells treated with 5 μM T-96, 9 ± 0.2% and 9.87 ± 0.21% in cells treated with 10 μM T-96(Figure 4A-B). Meanwhile, we observed that treatments with T-96 for 24 hours caused an obvious difference concerning the cancer cells' morphology. Together, these findings demonstrated that T-96 can elicit apoptosis in ESCC.

### Altered expression of apoptotic proteins in ESCC cells treated with T-96

**Figure 5:**
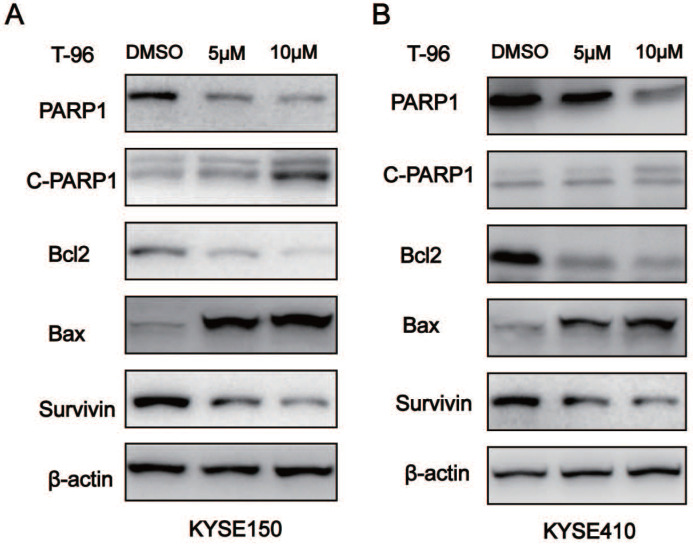
The effects of Demethylzeylasteral(T-96) on apoptosis related proteins. (A-B) Western blot was used to detect the expression of PARP1, cleaved PARP1 (C-PARP1), Bcl-2, Bax and Survivin. β-actin was used as a load control. β-actin was served as loading control.* *P* <0.05 ,** *P* <0.01 and *** P <0.001 as compared with the control group.

We further characterized T-96-induced apoptosis in the ESCC cells by analyzing the apoptotic proteins' expression. As indicated by western blot analysis, treatments with T-96 resulted in more Cleaved-PARP1 and Bax proteins, but decreased expression of Bcl2, Survivin and PARP1 in the cancer cells when compared with the control group (Figure 5). Furthermore, the ratio of Bax/Bcl2 increased in the experimental group. All these data provided more evidence that T-96 can induce apoptosis in esophageal cancer cells.

### T-96 inhibits EMT by regulating Wnt/β-catenin signaling pathway

**Figure 6:**
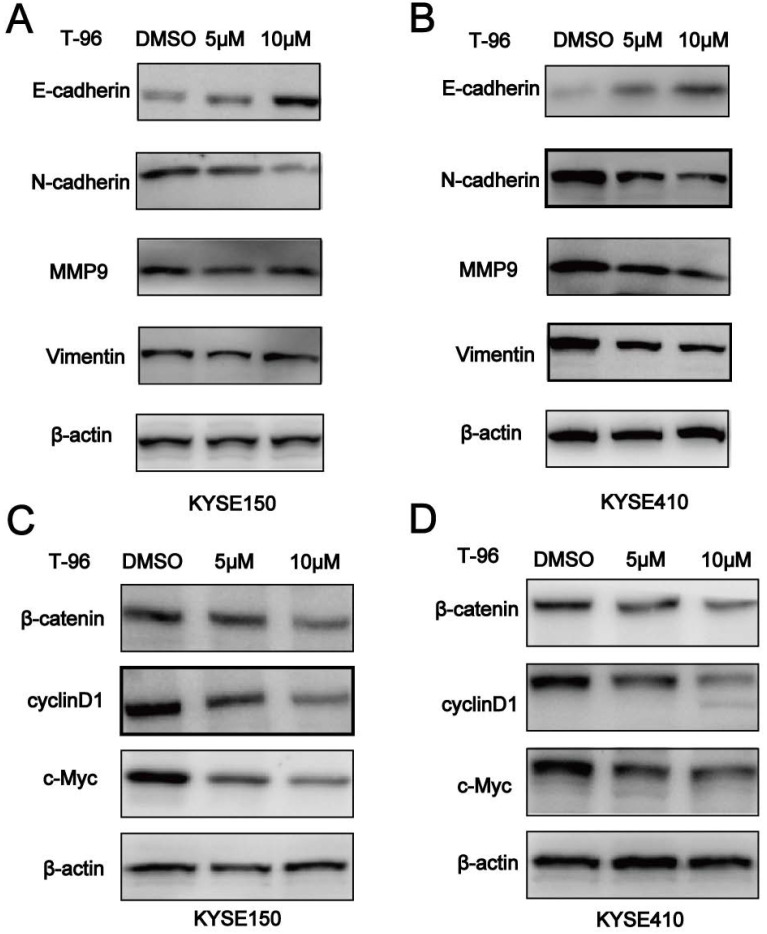
Demethylzeylasteral(T-96) inhibits EMT by regulating Wnt/β-catenin signaling pathway.( A-B) Western blot was used to detect the expression of E-cadherin, N-cadherin, Vimentin and MMP-9. (C-D) Western blot was used to detect the expression of β-catenin, cyclinD1 and c-Myc. β-actin was used as a load control. β-actin was served as loading control. Data represent the means ± SD of four independent experiments.* *P* <0.05, ** *P* <0.01 and *** P <0.001 as compared with the control group.

It is known that epithelial-mesenchymal-transition (EMT) is essential for tumor cells to migrate, and Wnt/β-catenin pathway should not be ignored in this process. Given the inhibitory effect of T-96 on ESCC cell migration, we set out to investigate whether EMT was altered in T-96-treated ESCC cells by examining the expression of EMT markers. According to Figure 6A-B, increased concentrations of T-96 resulted in E-cadherin's up-regulation as well as a significant reduction in the expression of Vimentin, N-cadherin and MMP-9 in the cancer cells treated with T-96 for 24 hours. T-96 can also affect the expression of EMT-related protein mRNA in ESCC cells (Supplementary Figure 1). Moreover, western blot analysis revealed a reduced level of β-catenin protein as well as a significant dose-dependent decrease in the expression of Wnt/β-catenin signaling downstream target genes cyclin D1, c-Myc and Wnt3a (Figure 6C-D, Supplementary Figure 2). Collectively, these data indicated that T-96 inhibits EMT by regulating Wnt/β-catenin pathway.

## Discussion

Domestically, esophageal cancer ranks fifth in incidence. Over 90% of esophageal cancers belong to squamous cell carcinoma 16. Patients with ESCC have a poor prognosis and do not respond to most conventional chemotherapy drugs 17. Increasing evidence has shown that natural small molecule drugs have the potential to become new compounds for cancer treatment 18, 19. Active as T-96 is, it came from Tripterygium wilfordii that exhibits various pharmacological effects, including metabolic modulation of hormones, immunosuppressive and antitumor effects 20-23. In this study, it is found that T-96 can inhibit the proliferation and migration of ESCC cells, and give rise to cell cycle arrest and apoptosis. Furthermore, T-96 inhibits EMT in the cancer cells by regulating Wnt/β-catenin pathway.

It is proved that T-96 from Tripterygium wilfordii with recognized anti-tumor activity effectively attenuated ESCC's proliferative capacity *in vitro*, and increased concentrations of the compound and prolonged time for treatment led to an inhibition in the cancer cell proliferation. T-96 can not only suppress the cancer cell growth but also the colony forming ability of ESCC. Notably, the colony forming ability of esophageal cancer cells was inversely proportional to the concentration of T-96 used in the experiments. Meanwhile, we observed that T-96 displays an inhibitory effect on the cancer cell migration. It is known that as an important biological feature of many cancers 24. Cancer metastasis makes up 90% of cancer mortality 25. T-96 is supposed to be have inhibition when breast, pancreatic, and glioma cells proliferate and migrate 6-8.

Generally, tumor cells are featured by abnormal cell cycle regulation, which is due to the structure and role that change cyclin and cyclin dependent kinases 26. According to relevant studies, lots of small molecular products extracted from natural drugs inhibit the proliferation of cancer cells 27. Similarly, in lots of cancers, such as pancreatic cancer, it is proved that T-96 has the same function 7. It lives up to our expectation that 48 hours after T-96 treatment, obvious G2/M phase arrest happened in KYSE150 and KYSE410 cells. It is known to all that the cell transformation from G2 to M phase is dominated by CyclinB1. G2/M serves as a DNA damage checkpoint that prevents cells with genomic DNA damage from entering M phase 28. As expected, Cyclin B1 protein and mRNA in KYSE150 and KYSE410 cells treated with T-96 decreased. T-96 inhibited the cell cycle and the proliferation of ESCC cells. A large number of studies have pointed out that apoptosis is a promising and has been adopted to screen and assess chemotherapy drugs 29, 30. Further research findings that apoptosis is well managed process in which dead cells are cleared without causing inflammation and damage to the surrounding cells. Here, we found that T-96 treatment to a large extent increased apoptotic cells, while down-regulated Bcl2, an anti-apoptotic factor. This finding was consistent with a recent report that T-96 can attenuate the pancreatic cancer cell proliferation and accelerate the cancer cell apoptosis.

Despite the observations that T-96 negatively regulates tumor cell proliferation and migration in most cancers, it remains to be investigated whether it affects EMT, thereby inhibiting tumor cell migration. Metastasis is the most important biological behavior of malignant cells, and EMT is crucial in the cancer invasion and metastasis 31, 32. It has been reported that many signaling pathways are implicated into EMT regulation. EMT is a complex process. It has been proved that Wnt/β-catenin signaling pathway is activated during EMT, while Wnt/β-catenin pathway inactivation can inhibit and reverse EMT 33, 34. Currently, the mRNA and protein levels of E-cadherin, N-cadherin, Vimentin, and MMP9 in the ESCC cells incubated with T-96 are studied. As expected, T-96 inhibited EMT in the cancer cells. The Wnt/β-catenin signaling should not be neglected in various biological processes and EMT regulation. Blocking Wnt/β-catenin signaling activities can inhibit EMT by inducing epithelial differentiation. Therefore, developing new drugs and effective approaches since inhibiting Wnt/β-catenin pathway and EMT is crucial for controlling cancer metastasis.

Wnt/β-catenin signaling pathway is important for the growth control of epithelial cells, and has been the pursuit of anti-cancer drugs development 35. Increasing evidence suggests that Wnt/β-catenin signaling is critical when managing stem cell-like characteristics and EMT in metastasis. Dysregulation of Wnt/β-catenin pathway should not be separated with different cancer, including esophageal cancer 36, 37. T-96 can inhibit the growth of clones and function in the regulation of cell proliferation, metastasis and metastasis pathways through regulating the expression of Wnt/β-catenin signaling responsive genes. β-catenin acts as a key indicator for cell fatalization and EMT in metastatic cancers. Moreover, it was related to drug resistance and recurrence in patients suffering from esophageal cancer 38-40. T-96 inhibits the expression of Wnt/β-catenin signaling downstream target genes, thus inhibiting Wnt/β-catenin signaling activities.

Here, we provided the first demonstration that T-96 has a significant inhibitory effect on ESCC proliferation, and treatment with this compound reversed the EMT of malignant ESCC. However, while the targets of T-96 have not yet been identified, the *in vivo* role of this compound needs to be further investigated. T-96 displays far fewer side effects than Tripterygium wilfordii and has little effect on the weight of nude mice. In addition, T-96 exhibits the same immunosuppressive and antitumor activities as other Tripterygium wilfordii monomers.While T-96 is less toxic than other monomers in Tripterygium wilfordii, this compound is 1000 times less cytotoxic than triptolide. Thus, the relative safety of T-96 is higher. Given that in comparison with triptolide,T-96 has different targets, further studies should be focused on the mechanism of action for identifying their targets.Besides,T-96 has low oral bioavailability with 4.2% of the absolute bioavailability 22. Therefore, development of new formulations or semi-synthetic derivatives for improving the bioavailability of T-96 will be beneficial for its possibly clinical application.

In summary, T-96 has inhibition when the ESCC cell proliferate and migrate. Moreover, it gives rise to the cancer cell cycle arrest and apoptosis through regulating Wnt/β-catenin signaling, suggesting a clinical potential for ESCC treatment.

## Supplementary Material

Supplementary figures.

## Figures and Tables

**Table 1:** Primer sequences used in this article

Gene	Forward (5'-3')	Reverse (5'-3')
MMP9	ATCCCCCAACCTTTACCA	TCAGAACCGACCTACAA
E-cadherin	CGAGAGCTACACGTTCACGG	GGGTGTCGAGGGAAAAATAGG
N-cadherin	TTTGATGGAGGTCTCCTAACAC	ACGTTTAACACGTTGGAAATGTG
Vimentin	GACAATGCGTCTCTGGCACGTCTT	TCCTCCGCCTCCTGCAGGTTCTT
β-actin	CTCCATCCTGGCCTCGCTGT	GCTGTCACCTTCACCGTTCC

## References

[1] Siegel RL, Miller KD, Jemal A (2019). Cancer statistics, 2019. CA: a cancer journal for clinicians.

[2] Yang H, Liu H, Chen Y, Zhu C, Fang W, Yu Z (2018). Neoadjuvant Chemoradiotherapy Followed by Surgery Versus Surgery Alone for Locally Advanced Squamous Cell Carcinoma of the Esophagus (NEOCRTEC5010): A Phase III Multicenter, Randomized, Open-Label Clinical Trial. Journal of clinical oncology: official journal of the American Society of Clinical Oncology.

[3] Mariette C, Markar SR, Dabakuyo-Yonli TS, Meunier B, Pezet D, Collet D (2019). Hybrid Minimally Invasive Esophagectomy for Esophageal Cancer. The New England journal of medicine.

[4] Chen Y, Ye J, Zhu Z, Zhao W, Zhou J, Wu C (2019). Comparing Paclitaxel Plus Fluorouracil Versus Cisplatin Plus Fluorouracil in Chemoradiotherapy for Locally Advanced Esophageal Squamous Cell Cancer: A Randomized, Multicenter, Phase III Clinical Trial. Journal of clinical oncology: official journal of the American Society of Clinical Oncology.

[5] Wang T, Shen F, Su S, Bai Y, Guo S, Yan H (2016). Comparative analysis of four terpenoids in root and cortex of Tripterygium wilfordii Radix by different drying methods. BMC complementary and alternative medicine.

[6] Li L, Ji Y, Fan J, Li F, Li Y, Wu M (2019). Demethylzeylasteral (T-96) inhibits triple-negative breast cancer invasion by blocking the canonical and non-canonical TGF-β signaling pathways. Naunyn-Schmiedeberg's archives of pharmacology.

[7] Wang F, Tian X, Zhang Z, Ma Y, Xie X, Liang J (2018). Demethylzeylasteral (ZST93) inhibits cell growth and enhances cell chemosensitivity to gemcitabine in human pancreatic cancer cells via apoptotic and autophagic pathways. International journal of cancer.

[8] Zhang K, Fu G, Pan G, Li C, Shen L, Hu R (2018). Demethylzeylasteral inhibits glioma growth by regulating the miR-30e-5p/MYBL2 axis. Cell death & disease.

[9] Zhao Y, He J, Li J, Peng X, Wang X, Dong Z (2017). Demethylzeylasteral inhibits cell proliferation and induces apoptosis through suppressing MCL1 in melanoma cells. Cell death & disease.

[10] Clevers H (2006). Wnt/beta-catenin signaling in development and disease. Cell.

[11] Yang S, Liu Y, Li MY, Ng CSH, Yang SL, Wang S (2017). FOXP3 promotes tumor growth and metastasis by activating Wnt/β-catenin signaling pathway and EMT in non-small cell lung cancer. Molecular cancer.

[12] Lee JM, Dedhar S, Kalluri R, Thompson EW (2006). The epithelial-mesenchymal transition: new insights in signaling, development, and disease. The Journal of cell biology.

[13] Stuelten CH, Parent CA, Montell DJ (2018). Cell motility in cancer invasion and metastasis: insights from simple model organisms. Nature reviews Cancer.

[14] Nusse R, Clevers H (2017). Wnt/β-Catenin Signaling, Disease, and Emerging Therapeutic Modalities. Cell.

[15] Zhao Z, Li L, Du P, Ma L, Zhang W, Zheng L (2019). Transcriptional Downregulation of miR-4306 serves as a New Therapeutic Target for Triple Negative Breast Cancer. Theranostics.

[16] White RE, Abnet CC, Mungatana CK, Dawsey SM (2002). Oesophageal cancer: a common malignancy in young people of Bomet District, Kenya. Lancet (London, England).

[17] Noordman BJ, Spaander MCW, Valkema R, Wijnhoven BPL, van Berge Henegouwen MI, Shapiro J (2018). Detection of residual disease after neoadjuvant chemoradiotherapy for oesophageal cancer (preSANO): a prospective multicentre, diagnostic cohort study. The Lancet Oncology.

[18] Huang MY, Zhang LL, Ding J, Lu JJ (2018). Anticancer drug discovery from Chinese medicinal herbs. Chinese medicine.

[19] Feng YL, Chen DQ, Vaziri ND, Guo Y, Zhao YY (2020). Small molecule inhibitors of epithelial-mesenchymal transition for the treatment of cancer and fibrosis. Medicinal research reviews.

[20] Wong KF, Yuan Y, Luk JM (2012). Tripterygium wilfordii bioactive compounds as anticancer and anti-inflammatory agents. Clinical and experimental pharmacology & physiology.

[21] Hu Q, Yang C, Wang Q, Zeng H, Qin W (2015). Demethylzeylasteral (T-96) Treatment Ameliorates Mice Lupus Nephritis Accompanied by Inhibiting Activation of NF-κB Pathway. PLoS One.

[22] Xu W, Lin Z, Yang C, Zhang Y, Wang G, Xu X (2009). Immunosuppressive effects of demethylzeylasteral in a rat kidney transplantation model. International immunopharmacology.

[23] Ushiro S, Ono M, Nakayama J, Fujiwara T, Komatsu Y, Sugimachi K (1997). New nortriterpenoid isolated from anti-rheumatoid arthritic plant, Tripterygium wilfordii, modulates tumor growth and neovascularization. International journal of cancer.

[24] Gupta GP, Massagué J (2006). Cancer metastasis: building a framework. Cell.

[25] Steeg PS (2006). Tumor metastasis: mechanistic insights and clinical challenges. Nature medicine.

[26] Wang Z, Fan M, Candas D, Zhang TQ, Qin L, Eldridge A (2014). Cyclin B1/Cdk1 coordinates mitochondrial respiration for cell-cycle G2/M progression. Developmental cell.

[27] Zheng K, He Z, Kitazato K, Wang Y (2019). Selective Autophagy Regulates Cell Cycle in Cancer Therapy. Theranostics.

[28] Petroni G, Formenti SC, Chen-Kiang S, Galluzzi L (2020). Immunomodulation by anticancer cell cycle inhibitors. Nature reviews Immunology.

[29] Tamm I, Schriever F, Dörken B (2001). Apoptosis: implications of basic research for clinical oncology. The Lancet Oncology.

[30] Burgess DJ (2013). Apoptosis: Refined and lethal. Nature reviews Cancer.

[31] Zhou J, Tan X, Tan Y, Li Q, Ma J, Wang G (2018). Mesenchymal Stem Cell Derived Exosomes in Cancer Progression, Metastasis and Drug Delivery: A Comprehensive Review. Journal of Cancer.

[32] Yang J, Antin P, Berx G, Blanpain C, Brabletz T, Bronner M (2020). Guidelines and definitions for research on epithelial-mesenchymal transition. Nature reviews Molecular cell biology.

[33] Cui Y, Zhang L, Wang W, Ma S, Liu H, Zang X (2019). Downregulation of nicotinamide N-methyltransferase inhibits migration and epithelial-mesenchymal transition of esophageal squamous cell carcinoma via Wnt/β-catenin pathway. Molecular and cellular biochemistry.

[34] Sinnberg T, Levesque MP, Krochmann J, Cheng PF, Ikenberg K, Meraz-Torres F (2018). Wnt-signaling enhances neural crest migration of melanoma cells and induces an invasive phenotype. Molecular cancer.

[35] Zhan T, Rindtorff N, Boutros M (2017). Wnt signaling in cancer. Oncogene.

[36] Wu Y, Yang Y, Xian YS (2019). HCRP1 inhibits cell proliferation and invasion and promotes chemosensitivity in esophageal squamous cell carcinoma. Chemico-biological interactions.

[37] Chen D, Li W, Liu S, Su Y, Han G, Xu C (2015). Interleukin-23 promotes the epithelial-mesenchymal transition of oesophageal carcinoma cells via the Wnt/β-catenin pathway. Scientific reports.

[38] Han L, Dai S, Li Z, Zhang C, Wei S, Zhao R (2019). Combination of the natural compound Periplocin and TRAIL induce esophageal squamous cell carcinoma apoptosis *in vitro* and *in vivo*: Implication in anticancer therapy. Journal of experimental & clinical cancer research: CR.

[39] Lin DC, Zhang Y, Pan QJ, Yang H, Shi ZZ, Xie ZH (2011). PLK1 Is transcriptionally activated by NF-κB during cell detachment and enhances anoikis resistance through inhibiting β-catenin degradation in esophageal squamous cell carcinoma. Clinical cancer research: an official journal of the American Association for Cancer Research.

[40] Gan SY, Zhong XY, Xie SM, Li SM, Peng H, Luo F (2010). Expression and significance of tumor drug resistance related proteins and beta-catenin in esophageal squamous cell carcinoma. Chinese journal of cancer.

