# Supplementary material for: Demethylzelasteral inhibits proliferation and EMT via repressing Wnt/β-catenin signaling in esophageal squamous cell carcinoma
Source: J Cancer. 2021 May 10;12(13):3967–75. doi: 10.7150/jca.45493 (PMC8176255; doi:10.7150/jca.45493)
Supplement: Supplementary file 1 — Supplementary figures. [file jcav12p3967s1.pdf]

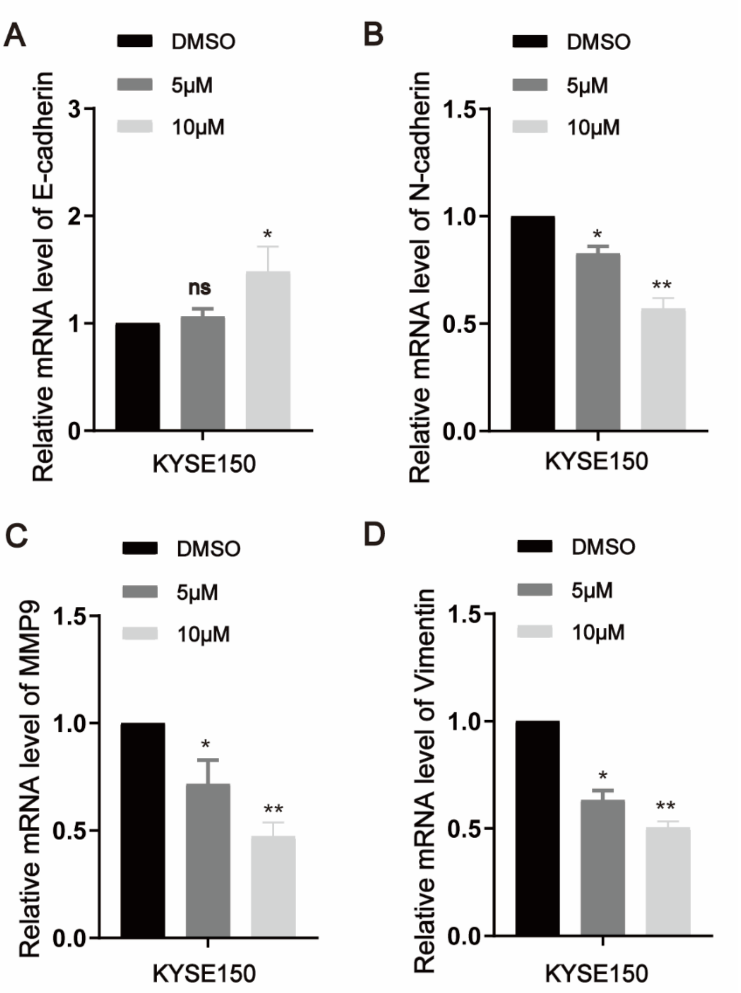

Supplementary Figure1. The mRNA levels of E-cadherin, N-cadherin, Vimentin and MMP9 were detected by qRT-PCR. (A-D) Relative mRNA expression of E-cadherin, N-cadherin, Vimentin and MMP9 in KYSE150 cells was analysed by quantitative real-time PCR andnormalized to β-actin mRNA expression. The data represent three independent experiments. ***P* < 0.01 and ****P* < 0.001 as compared to the control group.

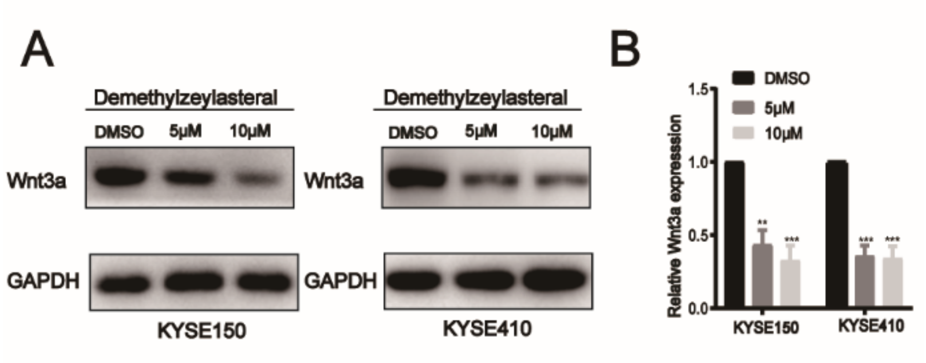

Supplementary Figure 2. The Wnt3a was detected by Western blot. (A) The protein levels of Wnt3a was measured by western blotting.(B) Bar graphs represent quantitative differences in the expression of integrin Wnt3a was used as a load control. GAPDH was served as loading control.***P* < 0.01 and ****P* < 0.001 as compared to the control group.
